# A synthesized view of the CSF-blood barrier and its surgical implications for aging disorders

**DOI:** 10.3389/fnagi.2024.1492449

**Published:** 2025-02-05

**Authors:** Birra Taha, Robert McGovern, Cornelius Lam

**Affiliations:** ^1^Department of Neurosurgery, University of Minnesota, Minneapolis, MN, United States; ^2^Minneapolis VA Health Care System, Veterans Health Administration, United States Department of Veterans Affairs, Minneapolis, MN, United States

**Keywords:** CSF (cerebrospinal fluid), hydrocephalus, Alzheimer’s disease, choroid plexus, endoscopic third ventriculostomy (ETV)

## Abstract

In this review, we explore the mechanisms of the blood-cerebrospinal fluid (CSF) barrier and CSF transport. We briefly review the mathematical framework for CSF transport as described by a set of well-studied partial differential equations. Moreover, we describe the major contributors of CSF flow through both diffusive and convective forces beginning at the molecular level and extending into macroscopic clinical observations. In addition, we review neurosurgical perspectives in understanding CSF outflow pathways. Finally, we discuss the implications of flow dysregulation in the context of neurodegenerative diseases and discuss the rising role of perivascular drainage pathways including glymphatics.

## Fluid flow as a dynamical system incorporating diffusion and convection

In understanding the movement of fluids, flow is generally modeled as a combination of diffusive and convective forces. The partial differential equation describing the convective-diffusive behaviors exists as a derivation of a more generalized equation of the continuity of any transport phenomenon, appropriately called the “continuity equation.” Cerebrospinal fluid (CSF) can be understood as a Newtonian fluid with incompressible flow as modeled by the Navier–Stokes equation (1). With CSF, with certain safe assumptions regarding flow behavior and fluid properties, the convective-diffusive equation (derived from the Navier–Stokes equation) can be drastically simplified to:

(1) $$\begin{array}{l} \rho \left(\frac{\partial u}{\partial t}+u\cdot \nabla u\right)=-\nabla p+\mu \nabla^{2}u\\ \nabla \cdot u=0 \end{array}$$

Where **u** represents the velocity vector of CSF flow, “ρ” represents the scalar pressure field, μ and ρ represent CSF viscosity and density, respectively. While numerous computational models of CSF flow rely on this equation, its limitations cannot be ignored. Firstly, a respiratory component to CSF flow is often not included in numerical models (Khani et al., 2020). Second, nonpulsatile contributions including from cilia (Siyahhan et al., 2014) are often difficult to quantify and are commonly neglected. The limitations can be extended to even questions regarding channel porosity (see Darcy’s Law) and irregular channel shape (Thomas, 2019), Convective flow in CSF is defined as a single directional flow of fluid from the choroid plexus (CP) until absorbed at some terminal end point (classically understood at the dural sinuses). A net hydrostatic gradient is understood to drive the convective forces moving CSF from the CP through to the ventricular space and beyond. Diffusive flow in this context refers to the bidirectional driving force of flow from compartments in the interstitial space, the choroid plexus, and the ventricular lumen. Disruptions in this equilibrium across compartments (whether an excess of production or decreased absorption) can lead to conditions such as hydrocephalus. Alternatively, without significant change in net flux, disruptions in clearance of toxic metabolites (i.e., fluid content) can lead to other types of pathologies such as Alzheimer’s disease.

## Current molecular understanding of fluid flow

Understanding the constituents of CSF is critical. Overall, CSF composition is quite similar to plasma (Tumani, 2015). Sodium, potassium, calcium, and glucose levels in CSF are generally slightly lower as compared to plasma. While chloride, magnesium and overall osmolality are slightly higher as compared to plasma. CSF constituency is a function of transport phenomena relying on active and passive transport mechanisms of organic and inorganic ions, and peptides.

Creation of CSF is formed by both the choroid plexus and non-choroidal sources (Lun et al., 2015). The ependymal lining has been known to contribute to CSF formation (Lun et al., 2015). However, extensive literature stretching centuries have primarily implicated the choroid plexus as the main contributor (Lun et al., 2015). CSF production occurs in a staged process through leaky endothelial cells at the CSF-blood interface and the regulated secretion across the choroid plexus epithelium (CPe) (MacAulay et al., 2022). Tightly regulated mechanisms transporting water, ions and organic substrates via paracellular and transmembranous pathways drive CSF formation (MacAulay et al., 2022). Beginning first within the leaky endothelium of the choroid plexus vasculature. Subsequently, at the basolateral membrane of the CPe, Na^+^, and Cl^-^ is carried intracellularly by transmembranous gradients utilizing symport (NA^+^/HCO^3-^) and antiport systems (Na^+^/H^+^ exchanger, Cl^-^HCO^3-^ exchanger) (Dual function of the choroid plexus, 2023; MacAulay et al., 2022). Along the basolateral surface, carbonic anhydrase plays a vital role in generation of HCO^3-^ for eventual intracellular uptake. Pharmacological targeting to limit HCO^3-^ and H^+^ generation via carbonic anhydrase inhibitors has a clear role in reduction of CSF production--albeit limited by systemic side effects (particularly at the level of the kidneys) (Brown et al., 2004).

The Na^+^/K^+^ ATPase serves as the primary catalyst for Na^+^ gradient creation through the extracellular transport of Na+ and intracellular transport of K+. In conjunction with the Na/K pump is a triple ion Na-K-2Cl- cotransporter on the apical surface. NKCC cotransporter has been a popular pharmacological target and a topic of investigation in neurodegeneration (Xu et al., 2021). These ion transporters generate electrochemical gradients which drive transmembranous ion channels on the basolateral surface.

After direct stimulation or indirect potentiating of postsynaptic neurons, clearance from the synaptic cleft is via several mechanisms including catabolism (Bjorefeldt et al., 2018). Intact mechanisms for neurotransmitter catabolite clearance are crucial in the maintenance of an ideal environment for action potential generation and propagation. On the apical surface of the choroid plexus epithelium, the organic anion transporter (OAT) system exists as an efflux mechanism for many neurotransmitter metabolites (Nigam et al., 2015). An OAT family-dependent efflux mechanism can occur directly through transport into the blood (or indirectly through the inhibition of efflux of other organic anions) (Nigam et al., 2015). For example, VMA interacts with OAT1/3 through direct efflux and its inhibition of efflux other organic anions (Alebouyeh et al., 2003). In contrast to melatonin and HVA, which show only indirect influence (Alebouyeh et al., 2003).

## Convectional driving forces

The blood-CSF interface plays host to a delicate balance of outward (plasma-facing) and inward (luminal-facing) convectional forces. Arterial pulsations delivering blood at a rate of roughly 3 mL/g/min transmit a pressure wave through the ventricular compartment (Vikner et al., 2024) and mitigate CSF dynamics (Mestre et al., 2018). Intracranial pressure (ICP), defined as the difference between the mean arterial pressure and the cerebral perfusion pressure, directly correlates with CSF production rate. Elevated blood pressures also likely contribute to CSF overproduction. In models of hypertension in rats, the blood-CSF barrier undergoes rapid changes in cellular machinery and its secretory capabilities (Al-Sarraf and Philip, 2003). A significantly more permeable blood-CSF interface facilitates pan-macromolecule transport and leads to unchecked ventricular expansion (Gonzalez-Marrero et al., 2022). Presumably, hypertensive-induced CSF hypersecretion occurs, at least in part, due to a larger arterial pulse wave and consequently greater pressure gradient (Al-Sarraf and Philip, 2003).

Aquaporins (AQP) (and its numerous isoforms) play an important role in the passive diffusion of water at the blood-CSF interface most densely represented on the apical surface (Municio et al., 2023). AQP1 (the most active isoform) controls the majority of water diffusion/permeability on the apical surface as confirmed by decreased CSF production and ICP reduction in AQP1^−/−^ mice (Trillo-Contreras et al., 2019). AQP1 regulating agents have been suggested as possible avenues for managing pathological states where CSF is over-produced (congenital hydrocephalus) or under-produced (neurodegenerative diseases, etc.) (Yamada, 2023). Structurally, at the blood-CSF barrier, tight junctions at the level of CPe tightly regulates paracellular flow. Claudin-2, a major tight junction protein linking CPe, has been shown to be associated with enhance paracellular water movement and Na + diffusion (MacAulay et al., 2022).

Opposing these inward forces are forces in the venous system. At the level of the arachnoid granulations, CSF absorption into systemic venous circulation relies on a net positive pressure gradient. Consequently, in settings of increased cerebral venous pressures (sinus stenosis, advanced congestive heart failure, etc.), decreased CSF absorption and consequently increased ICPs are common (Zhao et al., 2022).

## Classic concepts of CSF creation and removal

Traditional understanding of CSF creation relies on a method of secretion found in analogous secretory epithelia throughout the body. Osmotic gradients created by both active and passive methods through transmembranous players drive transcellular and paracellular water movement via aquaporins. At the level of the apical surface of the CP, the Na-K ATPase pump is a key driver for Na + secretion into the CSF and subsequent passive absorption into CPe on the basolateral surface from the interstitial fluid (MacAulay et al., 2022). However, the osmotic gradient is not required for CSF secretion (Khamesi et al., 2023).

Classic understanding points to convective and diffusive forces propelling CSF absorption through the ventriculo-cisternal system into the venous system (by the arachnoid villi) or via perivascular means (Proulx, 2021). Nerve roots also serve as a route for egress for CSF (Proulx, 2021).

A complete perspective of CSF flow is as a product of bidirectional forces as described in the diffusive-convective equation shown in equation (1). At steady state, net forces favor a model of CSF creation. This is seen as a summed contribution of flow from transcellular and paracellular pathways. However, perturbations, whether pathological or physiological, may reveal settings where aggregate forces favoring egress may transiently dominate–emphasizing the dynamic nature of the system (Bothwell et al., 2019). However, as the perturbation is felt system-wide, understanding this interplay will be dependent on the structural and molecular substrates, and their respective concentrations, at their interface.

Landmark papers utilizing perfusion studies in rabbits (Welch, 1963) and cats (Lorenzo and Snodgrass, 1972) confirmed the vital role of the CP. Morphological studies have identified varying levels of complexity of arachnoid villi between humans, non-human primates and lower animals (Takahashi et al., 1993; Yoshida et al., 1994). Nasal lymphatics have been shown as possible absorption sites around nerve roots within the cribriform plate in pigs and rats and appear at different stages of postnatal development (Koh et al., 2006). These findings of a robust extracranial lymphatic route were confirmed in sheep (Boulton et al., 1998). Lymphatic drainage appears to show species-to-species differences with sheep (Mollanji et al., 2001) and rat (Boulton et al., 1999) showing high rates of CSF absorption. These interspecies differences in CSF physiology as well as large variances in circulating volume, turnover rates play a large role in drug delivery considerations (Naseri Kouzehgarani et al., 2021).

## Surgical data

Surgical options exist in the role of CSF diversion in cases of hydrocephalus. Endoscopic third ventriculostomy is a procedure where a stoma is created at the floor of the third ventricle—allowing CSF outflow upstream of the cerebral aqueduct. Choroid plexus cauterization (CPC) uses electrocautery to obliterate choroid plexus. A ventriculoperitoneal shunt is a mechanical method for removal of CSF from within the ventricles to the intra-abdominal space via a catheter that exists under the skin. Owing to historical understanding of the role of the CP in CSF creation, removal of the choroid plexus cauterization was initially seen as a promising treatment following experiments in the early 20th century (Dandy, 1918). However, subsequent studies in the 20th century with CPC in the treatment of pediatric hydrocephalus expressed doubt in the success. This doubt was planted even earlier when Milhorat et al. showed CPC performed on rhesus monkeys showed a maximum of only 40% decrease in CSF production (Milhorat et al., 1971). Pioneering work by Warf et al beginning in the 2000s revived interest in the use of CPC in addition to ETV (using a flexible endoscope) for complete cauterization of the choroid plexus with mostly positive results in children under one year of age [10.3171/ped.2005.103.6.0475]. Specifically, results showed most etiologies stood to benefit from the addition of CPC, without any added negative effects on subsequent shunt failure risk after failed or abandoned ETV/CPC (10.3171/2012.9.peds1236), nor infection risk (10.3171/ped.2005.103.6.0475).

Through the growing use of minimally invasive neuroendoscopy, indications for the surgical treatment of hydrocephalus continue to grow. In post-hemorrhagic hydrocephalus (PHH), inflammatory debris has been shown to elicit a hypersecretory phenotype in the choroid plexus of rats relying on various inflammatory signaling molecules (Karimy et al., 2017)—challenging previous notions of a more obstructive phenotype (Chen et al., 2017). In PHH, endoscopic removal and lavage of hematoma debris has shown promise in avoiding VP shunts (Honeyman et al., 2022), and with improved developmental outcomes (Behrens et al., 2020). The hypothesis being early removal of intraventricular blood may prevent the formation of post-inflammatory membranes and the wider release of inflammatory byproducts necessitating permanent CSF diversion. Currently, neuroendoscopic lavage (NEL) has been limited to case series (Behrens et al., 2020; d’Arcangues et al., 2018; Dvalishvili et al., 2022; Tirado-Caballero et al., 2020) and technical papers regarding safe techniques (Tirado-Caballero et al., 2021). A recent randomized controlled trial for neuroendoscopic lavage recently showed promise in reducing time requiring temporizing draining measures and shortened hospital stays (Qu et al., 2024). As the evidence for NEL continues to grow, steps toward protocolization seek to remove the variability in technique and patient population to properly assess the most important outcome: short and long-term outcomes in reducing shunt dependence.

In settings where the obstruction is at the level of the foramen of Monro, unilateral hydrocephalus may develop (involving a single lateral ventricle) and eventually cause symptoms. Septum pellucidotomy is a procedure traditionally performed through an endoscope, where the surgeon cauterizes and creates an opening in the septum pellucidum—allowing communication between both lateral ventricles and flow through the contralateral foramen of Monro. Safe corridors for this approach have been well characterized. For complex pathology, advanced neuroendoscopic techniques continue to be developed extending beyond the third ventricle, including aqueductoplasty (Fritsch and Schroeder, 2013; Sansone and Iskandar, 2005), fourth ventriculostomy (Giannetti et al., 2011), and fourth ventricular outflow foraminoplasty (Shim et al., 2017).

## Aging disorders

In Alzheimer’s disease, dysfunction at the level of the BBB has been well described (Sweeney et al., 2018; Zenaro et al., 2017). The role of the BCSFB in pathogenesis, however, has had a less understood role (Farrall and Wardlaw, 2009). Contemporary understanding points to beta amyloid accumulation within the CP as the primary driver of its altered structure and function (González-Marrero et al., 2015). However, growing evidence has pointed to specific patterns of inflammation leading to BCSFB dysregulation through the identification of both serum and CSF chemokines (Kunis et al., 2013; Stopa et al., 2018). Histopathological evidence has implicated multiple inflammatory cascades involving CPe–leading to eventual microvascular damage and secondary fibrosis (Prineas et al., 2016). Comparing serum and CSF from patients with mild cognitive impairment and healthy controls, Ott et al. identified specific proteins with altered permeability across the BCSFB and their associated inflammatory markers (Ott et al., 2018). Modern imaging techniques also have the ability to complement these findings. Increased CP volume (determined from automated ventricular segmentation models) is positively correlated with disease severity and negatively correlated with total tau levels (Ota et al., 2023). Significant differences in permeability at the CP has also been captured by dynamic contrast-enhanced MRI in patients progressing from MCI to AD (Choi et al., 2022). Similarly in Parkinson’s disease (PD), a common neurodegenerative disorder characterized by focal dopaminergic losses in the substantia nigra and striatum, the CP appears to have an emerging role. Alpha-synuclein levels appear to show some degree of transport across the BCSFB in rat models (Bates and Zheng, 2014), but its exact mechanism of clearance is not well understood. However, similar to AD, neuroimaging studies have uncovered a negative association with CP volume and cognitive function in PD (Jeong et al., 2023). Glymphatic drainage dysregulation can also be captured by morphological changes in the CP–which may be part of a unifying story in both AD and PD (Buccellato et al., 2022). Aging itself may contribute to reduced efficacy in the glymphatic clearance of accumulated proteins (Kress et al., 2014).

## Newer concepts of CSF outflow

Understanding clearance pathways of the CSF has been a notorious problem now receiving renewed interest. Classic understanding attributes CSF outflow to projections of the arachnoid layer through the dura into the dural sinuses and lacunae was first rigorously proven in the late 19th century with Key and Retzius (1876). Modern concepts of CSF outflow acknowledge a wide range of outflow pathways. More recently, the discovery of dural lymphatic drainage has reignited interest in perivascular drainage pathways (Proulx, 2021). Meningeal/dural drainage was found in close proximity to the major dural sinuses and other major vascular pathways. Noninvasive confirmation of these pathways has been performed using MRI (Absinta et al., 2017). CSF outflow along cranial nerves has also been investigated using tracer studies (Albayram et al., 2022). For example, multiple tracer studies have shown SAS injections lead to uptake in surrounding orbital tissues (Killer et al., 2007; Shen et al., 1985). These studies have been recapitulated along multiple species both microscopically and macroscopically (Erlich et al., 1989). CSF outflow also occurs through nasal lymphatics via the cribriform plate (Spera et al., 2023). Lymphatic vessels are reached via perineural pathways along olfactory nerve fibers or even through direct access to the SAS (Walter et al., 2006).

## Glymphatics

A perivascular drainage pathway native to the CNS has origins in the scientific literature for several decades (Robin, 1859). The “glymphatic system” was reintroduced again in the 2010s where it was discovered the delicate conditions required to visualize the network (Iliff et al., 2012). Conceptual understanding of glymphatics relies on three sequential components: inflow along perivascular spaces surrounding arteries that penetrate the parenchyma, CSF diffusion throughout interstitial space, return from interstitial space into larger cisterns in subarachnoid space and ventricles from large-caliber veinage (Jessen et al., 2015). Limitations in post-mortem fixation techniques, combined with the low-pressure, easily collapsible nature of the network added to the controversy (Hablitz and Nedergaard, 2021). The glymphatic system encircles the brain vasculature and is considered to be ensheathed by astrocyte end-feet with variable coverage (Jessen et al., 2015).

The extent and orientation of the enclosed space has a direct influence on glymphatic fluid movement–as evidenced in models for hypoxia (Mestre et al., 2020) vessel tortuosity in aging (Thore et al., 2007), and even cortical spreading depression (Schain et al., 2017). Aquaporin4 (AQP4) has recently been shown as a key regulator of glymphatic fluid transport on the membrane of the astrocytic end-feet (Mestre et al., 2018). AQP4 knockout models have shown substantially reduced CSF tracer uptake in addition to significantly reduced clearance of numerous compounds (amyloid-beta (Iliff et al., 2012), apoE family (Achariyar et al., 2016), and adeno-associated viruses). Dysregulated clearance of amyloid-beta (Ab) has been seen in AQP4 deletions and accelerates Ab accumulation. These findings have been recapitulated in rodent models for “misplaced” AQP4 proteins (without perivascular presence via Snta1 deletion) (Amiry-Moghaddam et al., 2003). The implication of these results points to the glymphatic system as a potential new therapeutic targeting in AD, but also with Parkinson’s disease (Hoshi et al., 2017).

## Future directions

Just as many of the original experiments in understanding CSF flow were based in rudimentary neuroimaging techniques using tracers, breakthroughs in current dynamic neuroimaging methods are ushering a similar new wave of understanding of CSF dynamics (Mehta et al., 2022). Dynamic contrast-enhanced magnetic resonance imaging (MRI) (Iliff et al., 2013), diffusion weighted image (Harrison et al., 2018), two-photon laser (Iliff et al., 2012), phase contrast imaging (Brinker et al., 2014), and dynamic PET (de Leon et al., 2017) can delineate time-dependent pathways of CSF flow. The rise of noninvasive dynamic imaging, coupled with the increasing popularity of computer vision applications in medicine, has the potential to make major breakthroughs in understanding CSF physiology (Boster et al., 2023). Clinically, these noninvasive methods have a large potential role in identifying surgical candidates in numerous neurosurgical diseases. In Chiari 1 malformation, phase contrast MRI has the ability to capture the degree of CSF obstruction from tonsillar herniation (Mauer et al., 2011), the velocity of CSF through the aqueduct (Bateman and Brown, 2012), and CSF communication with intracranial arachnoid cysts (Yildiz et al., 2005).

Normal pressure hydrocephalus (NPH) is a disease characterized by a triad of dementia, urinary incontinence and gait instability. Phase contrast MRI has shown promise in detecting NPH patients (Tawfik et al., 2017). Accurate analysis of CSF flow metrics captured by phase contrast imaging has also shown significant promise as a marker for severity in patients with Idiopathic intracranial hypertension (IIH) (Belal et al., 2020).

## Conclusion

Significant work has been undertaken to understand the underpinnings of CSF dynamics--however, numerous unsolved questions still exist. Specifically, despite its relatively sparse representation in the literature, the blood-CSF interface plays a crucial and growing role in the regulation of CSF movement. New pathways of understanding CSF transport phenomenon, including glymphatics, appear to show promising results in understanding neurodegenerative diseases such as Alzheimer’s disease and Parkinson’s disease.
